# The Effectiveness of Dialectical Behavior Therapy (DBT) in Reducing Suicidal Tendencies (ST) in Adolescents with Bipolar Disorder

**DOI:** 10.1192/j.eurpsy.2025.773

**Published:** 2025-08-26

**Authors:** N. S. G. Abdelrasheed

**Affiliations:** 1 Department of Education; 2 Director of Centre for Student Counseling, Dhofar University, Salalah, Oman

## Abstract

**Introduction:**

Suicidal tendencies are among the most common problems faced by adolescents who encounter difficulties, issues, or challenges affecting various aspects of their psychological and social lives, often leading to high suicide rates. Bipolar disorder, various depressive disorders, substance abuse disorders, psychosis, and eating disorders are among the most common leading causes of suicide and suicidal tendencies among adolescents. Dialectical Behavior Therapy (DBT) is an integrative therapy employing a combination of techniques. DBT aims to assess the individual’s ability to regulate emotions, manage relationships effectively, tolerate distress, reduce maladaptive responses, and decrease impulsive and self-destructive behaviors.

**Objectives:**

The current study aims to investigate the effect of using Dialectical Behavior Therapy (DBT) techniques in reducing suicidal tendencies (ST) among adolescents of both genders who suffer from bipolar disorder. Additionally, to verify the sustained effectiveness of these techniques in reducing suicide risk among adolescents.

**Methods:**

A one-group experimental design was used. The study sample consisted of a (31) adolescents with high or moderate levels of suicidal tendencies who were diagnosed with bipolar disorder. Initially, the Bipolar Disorder Scale was used to identify adolescents exhibiting symptoms of the disorder for more than 6 months. This was followed by administering the Suicidal Tendencies Scale. Individuals with high or moderate suicidal tendencies scores on this scale were selected to participate in the study. They then underwent 23 sessions of psychotherapy based on DBT. After the completion of the therapy program, the Suicidal Tendencies Scale was administered again, and a third administration took place two months after the completion of the program. The research tools included a DBT-based therapy program consisting of 23 sessions, with an average of 3 sessions per week, each lasting 45-60 minutes.

**Results:**

The results indicated a significant improvement among adolescents after the completion of the program. Participants reported benefiting from the program, expressing reduced feelings of despair and increased hope and optimism about life. The adolescents’ scores on the Suicidal Tendencies Scale decreased after the program ended compared to their scores before the program. Additionally, their scores remained lower two months after the program ended compared to their pre-program scores. This indicates a positive impact of the counseling program in reducing suicidal tendencies among adolescents, as well as the sustained effectiveness of the program.

**Conclusions:**

Dialectical Behavior Therapy (DBT) is effective in reducing suicidal tendencies and can be applied effectively to a range of other psychological disorders. It is essential to manage suicidal tendencies among adolescents to help decrease suicide rates.

**Disclosure of Interest:**

None Declared

